# A new mouse model of Canavan leukodystrophy displays hearing impairment due to central nervous system dysmyelination

**DOI:** 10.1242/dmm.014605

**Published:** 2014-03-28

**Authors:** Marina R. Carpinelli, Anne K. Voss, Michael G. Manning, Ashwyn A. Perera, Anne A. Cooray, Benjamin T. Kile, Rachel A. Burt

**Affiliations:** 1 Murdoch Childrens Research Institute, 50 Flemington Road, Parkville, VIC 3052, Australia.; 2 The HEARing Cooperative Research Centre, 550 Swanston Street, University of Melbourne, VIC 3010, Australia.; 3 Walter and Eliza Hall Institute of Medical Research, 1G Royal Parade, Parkville, VIC 3052, Australia.; 4 Department of Medical Biology, University of Melbourne, Parkville, VIC 3010, Australia.; 5 Department of Genetics, University of Melbourne, Parkville, VIC 3010, Australia.

**Keywords:** Canavan disease, Aspa, Aspartoacylase, Leukodystrophy, ENU mutagenesis, Myelin

## Abstract

Canavan disease is a leukodystrophy caused by mutations in the *ASPA* gene. This gene encodes the enzyme that converts N-acetylaspartate into acetate and aspartic acid. In Canavan disease, spongiform encephalopathy of the brain causes progressive mental retardation, motor deficit and death. We have isolated a mouse with a novel ethylnitrosourea-induced mutation in *Aspa*. This mutant, named *deaf14*, carries a c.516T>A mutation that is predicted to cause a p.Y172X protein truncation. No full-length ASPA protein is produced in *deaf14* brain and there is extensive spongy degeneration. Interestingly, we found that *deaf14* mice have an attenuated startle in response to loud noise. The first auditory brainstem response peak has normal latency and amplitude but peaks II, III, IV and V have increased latency and decreased amplitude in *deaf14* mice. Our work reveals a hitherto unappreciated pathology in a mouse model of Canavan disease, implying that auditory brainstem response testing could be used in diagnosis and to monitor the progression of this disease.

## INTRODUCTION

Canavan disease is a leukodystrophy (disorder of the brain white matter) (Canavan, 1931). Affected babies seem normal at birth but at about 3 months of age start to display hypotonia, macrocephaly, mental retardation, blindness, seizures and spasticity (Matalon et al., 1995). Patients usually die at about 18 months of age (Toriello et al., 2004). Inheritance is autosomal recessive and disease incidence is very low except for in Ashkenazi Jews, where 1 in 57–65 individuals carry a mutant allele (Feigenbaum et al., 2004; Strom et al., 2004). Brain pathology includes spongy degeneration, fluid accumulation, vacuolation between myelin lamellae, and swollen astrocytes with distorted mitochondria (Adachi et al., 1973).

Canavan disease is caused by loss-of-function mutations in the gene encoding aspartoacylase (Kaul et al., 1993). This enzyme converts one of the most abundant amino acids in mammalian brain, N-acetylaspartic acid (NAA) (Tallan et al., 1956), into acetate and aspartic acid. The concentration of NAA is elevated in patient urine and blood (Matalon et al., 1988). Aspartoacylase expression in the brain is confined to oligodendrocytes (Madhavarao et al., 2004). The mechanistic link between the observed brain pathology and the absence of aspartoacylase is unclear. One proposal is that the acetate released from NAA hydrolysis is required for myelin synthesis by oligodendrocytes (Namboodiri et al., 2006). Another hypothesis is that accumulation of NAA in the brain leads to osmotic dysregulation and consequent astrocyte swelling and vacuole formation within the myelin sheath (Baslow, 2003).

Many studies have demonstrated deafness in animal models of dysmyelinopathy (Ito et al., 2004; Kanzaki et al., 1985; Kim et al., 2013; Naito et al., 1999; Ochikubo et al., 1992; Roncagliolo et al., 2000; Shah and Salamy, 1980; Zhou et al., 1995). Furthermore, pharmacological inhibition of myelination during rat development also causes deafness (Shah et al., 1978). In this paper we describe a novel mouse model for Canavan disease that was isolated during an ethylnitrosourea (ENU) mutagenesis screen. We demonstrate that this *Aspa* mutant mouse, named *deaf14*, is deaf because auditory signals do not transmit normally through its central nervous system (CNS). Our work reveals a hitherto unappreciated pathology in a mouse model of Canavan disease, suggesting that auditory brainstem response (ABR) testing could be used in the diagnosis of this disease.

## RESULTS

### *deaf14* mice have a nonsense mutation in *Aspa*

We undertook an ENU mutagenesis screen for hearing loss, in which we screened 697 third-generation (G_3_) mice in 40 pedigrees for low acoustic startle response (ASR). The G_1_ part of this screen has been described previously (Carpinelli et al., 2013). Control BALB/c mice consistently displayed a maximum startle amplitude above 200 mV (data not shown). Therefore, the criterion used to identify mice of interest was maximum startle amplitude below 200 mV. 25% of G_3_ mice in each pedigree were expected to display recessive phenotypes for mutations segregating in that pedigree. In one pedigree, seven of 26 G_3_ mice displayed a low ASR (supplementary material Fig. S1). Breeding tests confirmed that this phenotype was inherited in an autosomal-recessive fashion and the mutant strain was named *deaf14*.

**Fig. 1.**
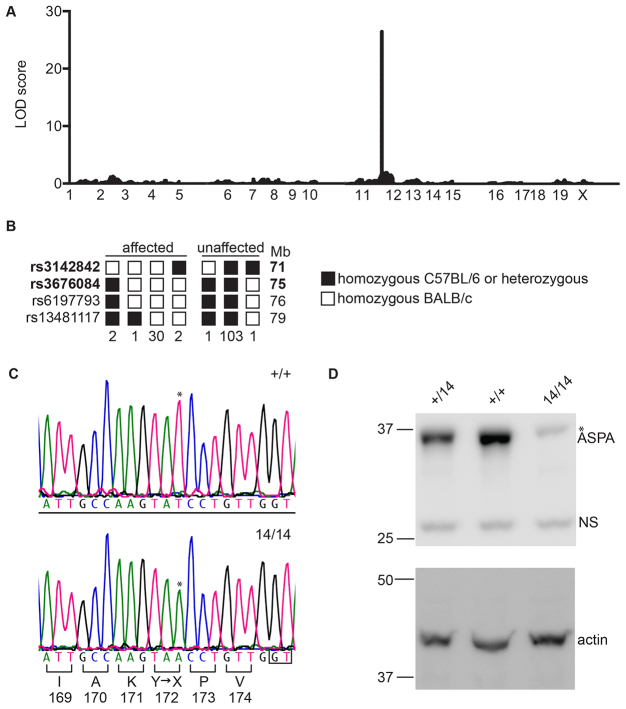
***deaf14* mice harbor a nonsense mutation in *Aspa*.** (A) Manhattan plot showing probability of linkage to *deaf14* locus across the genome. A LOD score above 3 is indicative of linkage. This was observed for a single peak on chromosome 11, indicating that the *deaf14* mutation is on this chromosome. (B) Haplotypes of 140 N_1_F_1_ mice for SNPs on chromosome 11. Mice were deemed affected or unaffected based on their click ABR. SNP identity is indicated on the left and chromosomal location is indicated on the right. The number of mice with each haplotype is indicated below. SNP locations were taken from Genome Reference Consortium Mouse Build 38 patch release 1 (GRCm38.p1). (C) DNA sequence of *Aspa* exon 3 in *+/+* (upper panel) and *deaf14/deaf14* mouse (lower panel). A c.516T>A mutation (*) is predicted to encode a p.Y172X premature termination of translation in the *deaf14/deaf14* mouse. (D) Western blot of p26 whole brain lysates probed with anti-ASPA (upper panel) and anti-actin (lower panel) antibodies. The upper panel shows a band at 35 kDa, the predicted molecular weight of ASPA, in *Aspa^+/+^* and *Aspa^+/deaf14^* brain that is not present in *Aspa^deaf14/deaf14^* brain. The faint band in the *Aspa^deaf14/deaf14^* lane (*) is of a slightly higher molecular weight and is also present in the two other lanes, albeit almost obscured by the ASPA band. This is likely to be a non-specific band. The lower panel demonstrates that equal amounts of protein were loaded in each lane. NS, non-specific band; 14, *deaf14*.

Genomic DNA from a female *deaf14/deaf14* mouse was subjected to exome enrichment and massively parallel DNA sequencing. 89.3% of the consensus coding sequence (CCDS) exome was sequenced at least fourfold and the average depth of sequencing was 58-fold. 144 single-nucleotide variants (SNVs) were recovered, 11 of which were homozygous. In order to determine which SNV was causative of the *deaf14* phenotype, a meiotic mapping cross was set up. A BALB/c*^deaf14/deaf14^* founder mouse was crossed to a C57BL6^+/+^ mouse and the offspring intercrossed. The resulting N_1_F_1_ offspring were ABR-tested and sacrificed for liver DNA isolation. DNA samples from 15 affected and 15 unaffected mice were genotyped at 660 single-nucleotide polymorphisms (SNPs) spaced at 5 Mb intervals across the genome. Calculation of log of the odds (LOD) scores revealed that the *deaf14* mutation was linked to chromosome 11 (Fig. 1A). This chromosome contained four homozygous SNVs, one in each of the *Aspa*, *Sez6*, *Thoc4* and *Abca9* genes. *Aspa* and *Sez6* were good candidates for carrying the *deaf14* causative mutation because *Aspa^−/−^* mice (Matalon et al., 2000) and *Sez6^−/−^* mice (Gunnersen et al., 2007) exhibit nervous-system defects. In order to determine whether *Aspa* carried the causative mutation, we undertook finer-resolution meiotic mapping around its location at 73 Mb. 140 N_1_F_1_ mice were genotyped for SNPs between 71 and 79 Mb (Fig. 1B). A region between 71 and 75 Mb was homozygous BALB/c in all affected mice and heterozygous or homozygous C57BL/6 in all unaffected mice. This showed that the *deaf14* mutation was in this interval. This excluded the *Sez6*, *Thoc4* and *Abca9* SNVs from being causative of hearing loss in *deaf14* mice, because they were outside this chromosomal region. The *Aspa* SNV was confirmed by Sanger DNA sequencing to be a c.516T>A mutation that is predicted to cause a p.Y172X protein truncation (Fig. 1C). Western blotting showed that full-length aspartoacylase was detected in brain of *Aspa^+/+^* and *Aspa^+/deaf14^* but not *Aspa^deaf14/deaf14^* mice at 26 days of age (Fig. 1D). A faint band was visible in all lanes at a slightly higher molecular weight than ASPA and was likely to be non-specific. Truncated protein was not detected, suggesting that *Aspa^deaf14^* is a null allele with respect to protein production.

**TRANSLATIONAL IMPACT**

**Clinical issue**

Canavan disease is a leukodystrophy – a disease characterized by degeneration of white matter of the brain – that is caused by recessive mutations in the *ASPA* gene. This gene encodes an enzyme, aspartoacylase, which is required for normal myelination of the brain. Symptoms of the disease include floppiness, a large head, mental retardation, blindness, seizures and spasticity. The onset of these symptoms can be as early as 3 months of age, and affected individuals usually die from progressive brain degeneration during childhood. Several studies have demonstrated a link between dysmyelination and hearing loss in animal models. Furthermore, deafness has been described in some individuals with Canavan disease. However, a definitive association between this phenotype and the disease has not been made to date.

**Results**

In this study, the authors performed an ENU mutagenesis screen for hearing loss, and isolated a mouse with a previously uncharacterized null mutation in *Aspa*. The mutant strain, named *deaf14*, was found not to startle in response to loud noise. The nerve leading from the ear to the brain (the auditory nerve) fired normally in this mouse; however, the brain neurons fired more slowly than normal and the sound signal dissipated. This indicates that auditory signal propagation is unaffected in the auditory nerve but impaired in the CNS. In line with this, *deaf14* mice showed extensive degeneration in the brain but not in the ear. These mice also displayed impairments in motor coordination.

**Implications and future directions**

This work indicates that hearing loss in *deaf14* mice is caused by defects in the CNS, resulting in an abnormal auditory brainstem response (ABR). This finding implies that deafness is linked with mutations in *Aspa* in Canavan disease. The ABR test is a non-invasive test that is currently used to screen newborn babies for hearing loss. In light of the present findings, this test could potentially be used as a simple and effective way to diagnose Canavan disease early on, allowing disease progression to be monitored prior to symptom-onset at 3 months. This report also provides a novel mouse model that could be used to investigate the molecular mechanisms of Canavan disease.

### *deaf14* mice have little acoustic startle response

**Fig. 2.**
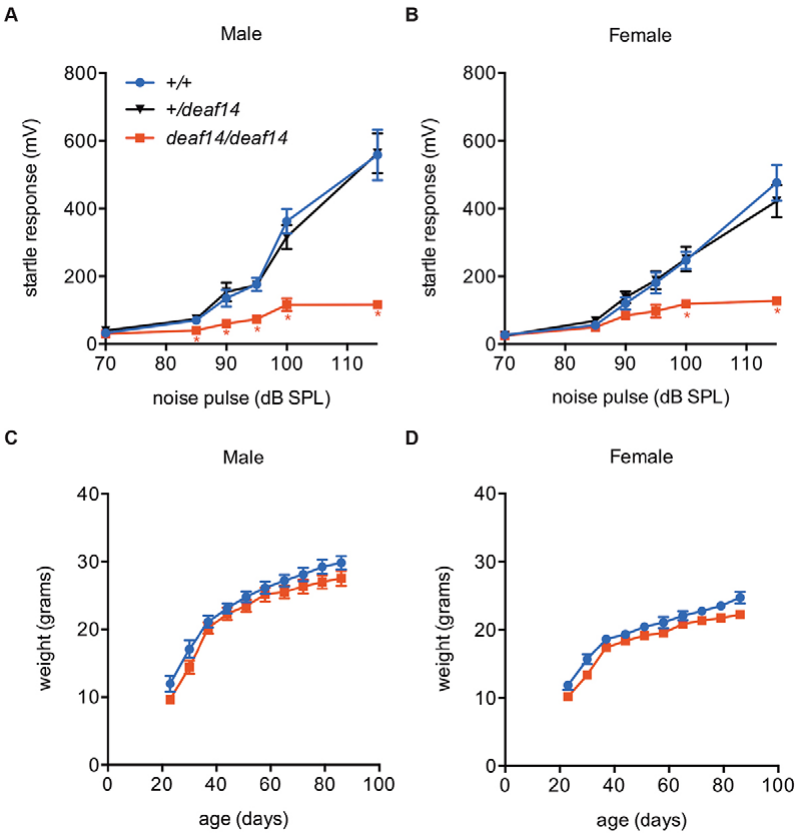
***deaf14* mice have a low acoustic startle response.** Startle response of (A) male and (B) female mice to white-noise pulses between 85 and 115 dB SPL. *Aspa^deaf14/deaf14^* mice had a lower magnitude startle response than *Aspa^+/+^* or *Aspa^+/deaf14^* mice. *n*=10 mice per group. * indicates a significant difference (*P*<0.05) in one-way ANOVA and Dunnett’s multiple comparison test versus *Aspa^+/+^*. Weights of (C) male and (D) female mice at 3 to 12 weeks of age. Mean *Aspa^deaf14/deaf14^* body weights were not significantly different from *Aspa^+/+^* mean body weights. A cut-off for statistical significance of *P*<0.01 was used to adjust for multiple testing. *n*=10 mice per group. Bars=s.e.m.

In order to determine the average startle response in *deaf14* mice, ASR was measured in mice that had been genotyped for the *deaf14* mutation. *Deaf14* mice startled to a lesser degree than wild-type littermates in response to white-noise pulses up to 115 dB sound pressure level (SPL) (Fig. 2A,B). This suggests that auditory signals are not reaching the parts of the brain where behavioral response is generated. Alternatively, motor deficit could cause the failure to startle. A low body weight could potentially lead to a spurious low startle response because of reduced accelerometer readings by the SR-LAB equipment. However, *deaf14* male and female mice were not significantly smaller than wild-type littermates (Fig. 2C,D). Thus, the low magnitude of startle response detected in these mice was not caused by low body weight.

### *deaf14* mice have an abnormal auditory brainstem response

**Fig. 3.**
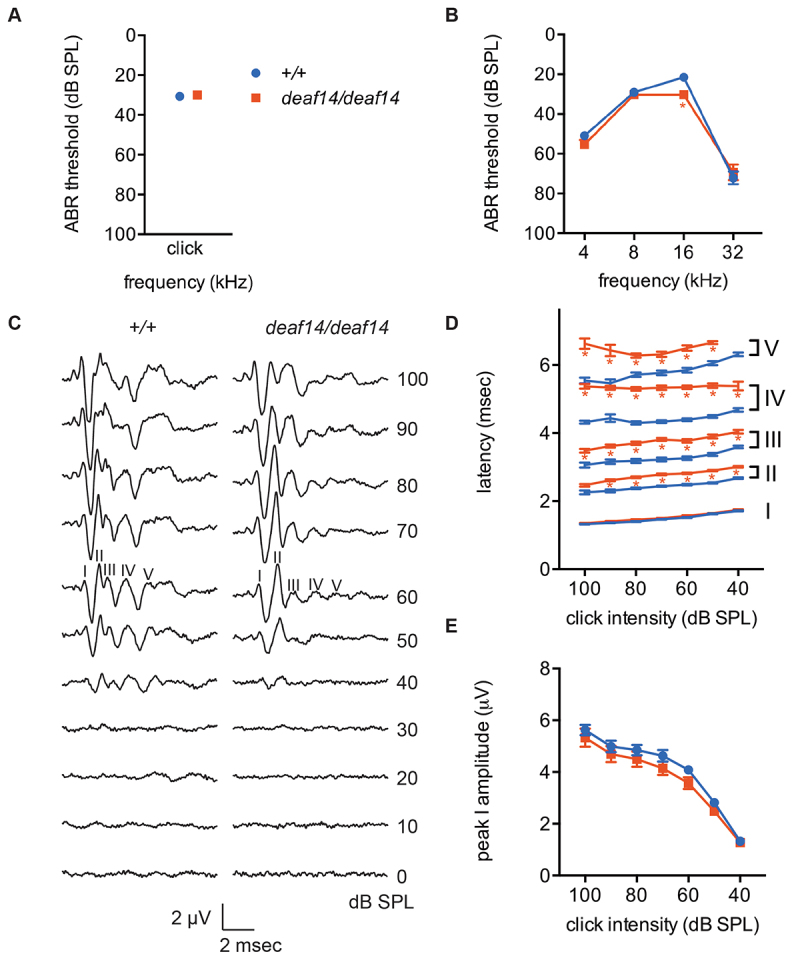
***deaf14* mice have abnormal auditory brainstem responses.** (A) Click and (B) pure-tone ABR thresholds at 8 weeks of age. *Aspa^deaf14/deaf14^* mice had an 8 dB SPL elevation in mean threshold at 16 kHz. (C) The shape of the click ABR was abnormal in *Aspa^deaf14/deaf14^* mice. Peaks are labeled at 60 dB SPL, where they are most easily distinguished. Note the reduced amplitude of later peaks at 50 dB SPL. (D) Latency of the first five click ABR peaks. Peaks II, III, IV and V had increased latency in *Aspa^deaf14/deaf14^* mice. (E) Growth function analysis of click ABR peak I. There was no difference between genotypes in the rate at which peak amplitude increased with stimulus intensity. *n*=8 female and 8 male mice per group. Bars=s.e.m. *P≤0.001 versus *Aspa^+/+^*.

ABR testing measures specific responses to auditory stimuli extracted from overall electrical activity in the brain. The ABR was measured in *deaf14* mice in order to determine whether their failure to startle was due to deafness. In mice, ABR peak I represents the signal from the auditory nerve, peak II the cochlear nucleus, peak III the superior olivary complex, peak IV the lateral lemniscus and peak V the inferior colliculus (Henry, 1979). *deaf14* mice had normal ABR thresholds in response to mixed frequency (click) stimuli (Fig. 3A). Thresholds to single-frequency stimuli were normal except for at 16 kHz, where the mean mutant threshold was 8 dB SPL above wild type (Fig. 3B). In our experience, a small elevation in ABR threshold is not sufficient to cause a low ASR. Furthermore, the ASR is elicited by mixed-frequency ‘white’ noise, not a 16 kHz pure tone. The shape of the ABR in *deaf14* mice was abnormal (Fig. 3C). Although peak I seemed normal, peaks II-V were reduced in size or absent. This is particularly evident at 50 dB SPL in Fig. 3C. ABR analysis revealed that the latency of peak I was normal in *deaf14* mice but peaks II, III, IV and V had longer latency than in wild-type littermates (Fig. 3D). This suggests that transition of the auditory signal through the CNS is abnormal in *deaf14* mice. Growth function analysis revealed that peak I increased in amplitude with signal intensity at a normal rate in *deaf14* mice (Fig. 3E). This indicates that the auditory nerve, which generates peak I, is able to function in the absence of aspartoacylase.

### *deaf14* brain displays spongy encephalopathy

**Fig. 4.**
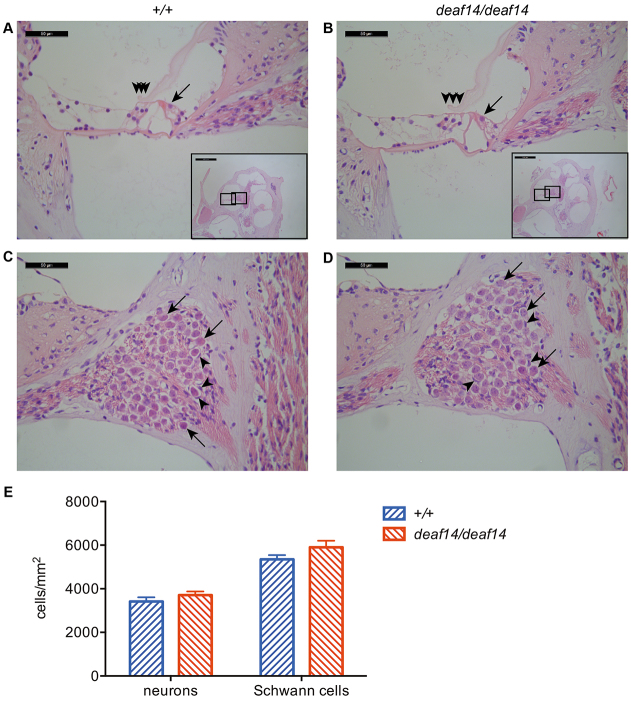
***deaf14* cochlea is histologically normal.** Mid-modiolar sections of *Aspa^+/+^* (A,C) and *Aspa^deaf14/deaf14^* (B,D) cochleae stained with hematoxylin and eosin. Insets show location of higher-power images in the middle cochlear turn. (A,B) One row of inner hair cells (arrow) and three rows of outer hair cells (arrowheads) were visible in wild-type and *deaf14* cochleae. (C,D) Neurons (arrows) and Schwann cells (arrowheads) in Rosenthal’s canal had normal morphology. (E) Neuron and Schwann cell densities were normal in *deaf14* mice (*P*>0.05). *n*=10–12 cochleae per genotype. Mice were 8 weeks of age. Scale bars: 50 μm in A–D and 500 μm in insets.

**Fig. 5.**
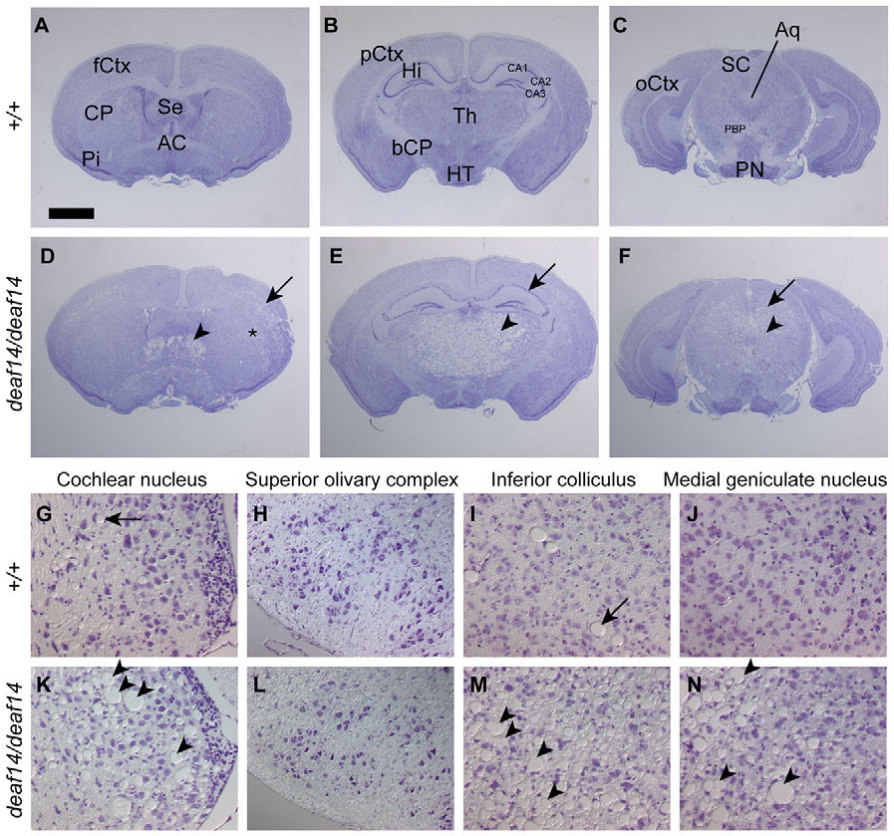
***deaf14* brain is extensively vacuolated.** Cresyl-violet-stained serial sections of immersion-fixed (A–F) or perfusion-fixed (G–N) brains of 10- to 12-week-old *Aspa^+/+^* (A–C,G–J) and *Aspa^deaf14/deaf14^* (D–F,K–N) mice. (A–F) Overview at the level of the frontal (A,D), parietal (B,E) and occipital cortex (C,F). Note the extensive vacuolization in layer V of the frontal cortex (arrow, D), the septal nuclei (arrowhead, D) and dorsolateral aspects of the caudate-putamen (asterix, D), the thalamus (arrowhead, E), the pyramidal layer of the hippocampus (arrow, E), the intermediate layers of the superior colliculi (arrow, F) and the peri-aqueductal region (arrowhead, F). (G–N) The auditory pathway, including the cochlear nucleus (G,K), the superior olivary complex (H,L), the inferior colliculus (I,M) and the medial geniculate nucleus (J,N). Note the extensive vacuolization (arrowheads, K,M,N) and the occasional distended blood vessel in the control (arrow, G–J). AC, anterior commissure; Aq, aqueduct; bCP, basal cerebral peduncle; CA1, 2, 3, fields of the hippocampus; fCtx, pCtx, oCtx, frontal, parietal and occipital cortex; CP, caudate putamen; Hi, hippocampus; HT, hypothalamus; PBP, parabrachial pigmented nuclei; Pi, piriform cortex; PN, pontine nuclei; SC, superior colliculus; Se, septum; Th thalamus. Scale bar in A: 1.4 mm in A-F and 92 μm in G–N.

The cochlea was examined in order to determine whether abnormalities in this structure were responsible for the abnormal ABR in *deaf14* mice. The *deaf14* cochlea proved indistinguishable from wild type, with three rows of outer hair cells and one row of inner hair cells visible in the middle turn (Fig. 4A,B). The neurons and Schwann cells making up the auditory nerve had normal morphology (Fig. 4C,D) and density (Fig. 4E). Next, the brain was examined for pathological abnormalities that could give rise to an abnormal ABR. The *deaf14* brain was grossly abnormal, with widely distributed spongiform encephalopathy and extensive vacuolation (Fig. 5). The affected areas from rostral to caudal were layer V of the frontal and anterior parietal cerebral cortex, the dorsolateral aspects of the caudate-putamen, the lateral septal nuclei, the thalamus, the pyramidal cell layer of CA1, CA2 and CA3 regions of the hippocampus, but not the granule cell layer of the dentate gyrus, the mammillary and supramammilary nuclei, anterior pretectal nuclei, suprageniculate thalamic nuclei, medial geniculate nuclei, intermediate layers of the superior colliculi, periaqueductal nuclei, inferior colliculi, ventral cochlear nuclei, vestibular nuclei, parabrachial nuclei, dorsal tegmental nuclei, and cerebellar white matter. The cerebellar Purkinje cell layer was affected in some areas.

### *deaf14* mice have impaired motor co-ordination

**Fig. 6.**
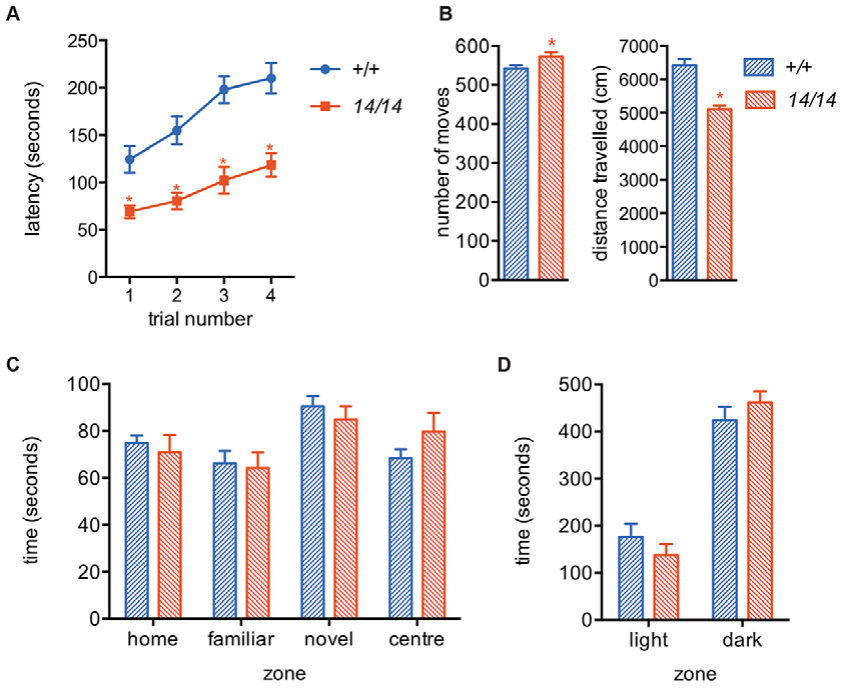
***deaf14* mice have impaired motor coordination.** (A) *deaf14* mice fell off a rotating rod before wild-type mice in four consecutive trials. (B) *deaf14* mice made more moves but travelled less distance than wild-type mice in an open field, suggesting ataxia. (C) *deaf14* mice were indistinguishable from wild-type mice in the Y-maze, which measures memory, and (D) the light dark test, which measures anxiety. *n*=9–10 mice per group. All mice were 9- to 10-weeks of age. *14*, *deaf14*. *P<0.05 versus *Aspa^+/+^*.

*deaf14* mice are viable to at least 350 days of age and by the age of 280 days developed a Parkinson’s disease-like tremor that was not present in age-matched wild-type mice (data not shown). In order to determine whether younger mice also had behavioral abnormalities, in particular motor impairment that could lead to a low ASR, behavioral testing was carried out at 9–10 weeks of age. *deaf14* mice had a shorter latency to fall off a rotating rod than wild-type mice (Fig. 6A), indicating impaired motor coordination. In the open-field test, *deaf14* mice made more moves but covered less distance than wild-type mice (Fig. 6B). This suggested that *deaf14* mice have an ataxic gait. *deaf14* mice displayed normal behavior in the Y-maze (Fig. 6C), which measures learning, and in the light-dark test (Fig. 6D), which measures anxiety. These results showed that impaired motor coordination in *deaf14* mice could be the cause of their low ASR.

## DISCUSSION

The first peak of the ABR had normal latency in *deaf14* mice and auditory neurons appeared normal on histological examination of the cochlea. However, latencies of peaks II, III, IV and V were increased in *deaf14* mice and the brain had extensive spongiform encephalopathy. This indicates that the auditory signal is able to propagate along the auditory nerve but does not propagate normally through the CNS. In *shiverer* mice, in which dysmyelination is confined to the CNS, the latency of ABR peak I is not significantly increased (Kanzaki et al., 1985), whereas, in *quaking viable* and *trembler* mice, with peripheral nervous system (PNS) dysmyelination, ABR peak I latency is increased (Shah and Salamy, 1980; Zhou et al., 1995). The *deaf14* ABR phenotype is similar to that of *shiverer*, supporting the conclusion that the CNS is the site of the defect causing deafness in these mice.

One rat and three mouse models of Canavan disease have been previously described (Kitada et al., 2000; Matalon et al., 2000; Mersmann et al., 2011; Traka et al., 2008) (Table 1). All carry null alleles of *Aspa. Aspa^deaf14^* is also likely to be a null allele, because (1) the single-nucleotide substitution causes a conversion of codon 172 to a stop codon, (2) no truncated protein was detected in p26 brain and (3) the *Aspa^deaf14/deaf14^* mutant phenotype is similar to that of *Aspa* null mutant mice (Mersmann et al., 2011). The Human Gene Mutation Database (www.hgmd.org) lists 77 mutations in *ASPA* (Stenson et al., 2014). The *Aspa^deaf14^* mutation lies in the region encoding Y172, which is orthologous to Y173 in human ASPA. A truncating mutation in codon 184 has been observed in a patient with Canavan disease (Zeng et al., 2002), providing further evidence that a nonsense mutation in this region of the gene disrupts function. The phenotype of *deaf14* mice is similar to that of the *Aspa^nur7^* (Traka et al., 2008) and *Aspa^lacZ^* (Mersmann et al., 2011) mutants but less severe than the *Aspa^Tm1Mata^* (knockout) strain, which displays ataxia and runting of greater severity (Matalon et al., 2000). This could be due the *Aspa^Tm1Mata^* mutation being on a 129S5/SvEvBrd genetic background and the *Aspa^deaf14^* mutation being on a BALB/c background. Alternatively, the difference could be due to the *neo* insertion in the knockout allele, which might affect expression of neighboring genes.

**Table 1.**
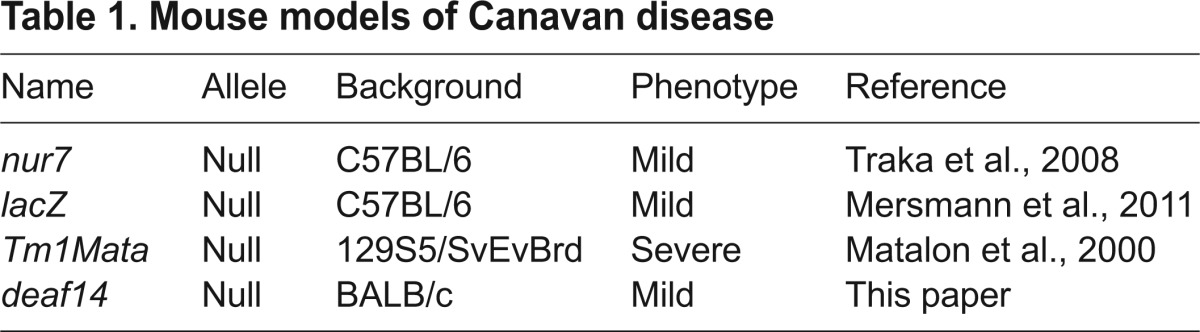
Mouse models of Canavan disease

*deaf14* mice were viable to at least 350 days of age and by the age of 280 days developed a Parkinson’s disease-like tremor that was not present in age-matched wild-type mice (data not shown). This indicated that the disease is progressive in mice, as it is in humans. It is of interest that aspartoacylase activity is required for survival past childhood in humans but not in mice. Given the progressive pathogenesis of the phenotype, the survival of mice to adulthood might be due to the shorter infancy, adolescence and overall lifespan of mice as compared to humans.

Deafness has been observed in individuals with Canavan disease (Toriello et al., 2004), but the only reference to this in the primary literature is of two Canavan disease siblings who were found to have an absent organ of Corti upon autopsy (Ishiyama et al., 2003). Because these individuals were from the same family, this abnormality might have been due to another deafness mutation segregating in this family. *deaf14* is the first animal model of Canavan disease to demonstrate deafness and ABR abnormalities. Furthermore, it is the first to indicate that humans with Canavan disease might have an abnormal ABR. If this is the case, then ABR testing might be useful for diagnosis and to monitor the progression of disease, particularly in children with a mild presentation (Yalcinkaya et al., 2005). In a similar manner, increased latency and decreased amplitude of ABR peaks have been used as criteria in the diagnosis of multiple sclerosis (Peyvandi et al., 2010; Sand et al., 2013). ABR testing is already used in universal newborn hearing screening (Mason and Herrmann, 1998) and is a rapid, inexpensive and non-invasive test. Given that other symptoms of Canavan disease are not detected until 3 months of age, inclusion of abnormal ABR in the diagnostic criteria for Canavan disease could aid in the early diagnosis of affected individuals, particularly those with the mild form.

## MATERIALS AND METHODS

### Mice

Mice were maintained at the Murdoch Childrens Research Institute (MCRI). Animals were group-housed in IVCD blue line micro-isolator cages (Tecniplast, Buguggiate, VA, Italy) on a 14-hour light/10-hour dark cycle. Animals were fed Barastoc mouse breeder cubes (Ridley AgriProducts, Melbourne, VIC, Australia) and water *ad libitum*. Mice underwent behavioral testing at the Melbourne Brain Centre (University of Melbourne, Parkville, VIC, Australia). All experiments involving animals were approved by the MCRI animal ethics committee (application number A726) and conformed to the Australian Code of Practice for the Care and Use of Animals for Scientific Purposes, 8th edition, 2013.

### Mutagenesis screen

Male BALB/c mice were intraperitoneally injected with 75 mg/kg body weight ethylnitrosourea (ENU, Sigma-Aldrich, Castle Hill, NSW, Australia) weekly for 3 weeks as described (Bode, 1984). Treated males were rested for 12 weeks before mating with untreated BALB/c females to produce G_1_ progeny. G_1_ mice from different fathers were intercrossed to produce G_2_ mice, which were brother-sister mated to produce G_3_ progeny. Under this breeding scheme, 25% of G_3_ mice in each pedigree will display a phenotype resulting from a recessive mutation inherited from their G_1_ grandparent. G_3_ mice were screened for deafness using ASR testing. The *deaf14* founder mouse was crossed to BALB/c for five generations to remove other ENU-induced mutations. *+/deaf14* mice were then intercrossed to generate N_5_F_1_ mice for phenotypic analysis.

### Acoustic startle response

ASR was measured using an SR-LAB system (San Diego Instruments, San Diego, CA). Each mouse was restrained in a Perspex chamber and acclimatized to background white noise of 70 dB SPL for 1 minute. Fifty-six trials were presented in pseudorandom order and separated by intervals of 3–8 seconds. Trials included background noise alone and 40 ms white-noise pulses of 85, 90, 95, 100 and 115 dB SPL. The average ASR for each stimulus was plotted against sound intensity using Prism 6 for Mac OS X software (GraphPad Software Inc., La Jolla, CA).

### Auditory brainstem response

Mice were anesthetized by intraperitoneal injection of 100 mg/kg body weight ketamine and 20 mg/kg body weight xylazine, and their eyes were moistened with Lacri-Lube. Body temperature was maintained with a 37°C heat pad inside a custom-made faraday chamber. The faraday chamber was placed inside a sound attenuation cabinet, the Habitest isolation cubicle model H10-24A (Coulbourn Instruments, Whitehall, PA). A magnetic speaker (Tucker Davis Technologies, Alachua, FL) was placed 10 cm from the left pinna and computer-generated clicks and pure-tone stimuli of 4, 8, 16 and 32 kHz were presented with maximum intensities of 100 dB SPL. ABRs were recorded differentially using subdermal needle electrodes (S06666-0, Rochester Electro-Medical, Inc., Lutz, FL). These were positioned at the vertex of the skull (+ve) and on the left cheek (−ve) with a ground on the hind left leg. ABRs were averaged over 512 repetitions of the stimulus. The amplitude and latency of ABR peaks I-V were determined using BioSig software (Tucker Davis Technologies). The ABR threshold was defined as the lowest intensity stimulus that reproducibly elicited an ABR.

### Meiotic mapping

A BALB/c*^deaf14/deaf14^* mouse was crossed to a C57BL/6^+/+^ mouse to generate N_1_ offspring, which were intercrossed to produce 140 N_1_F_1_ offspring. These mice were ABR-tested at 8 weeks of age and genomic DNA was extracted as described (Laird et al., 1991). Genomic DNA samples of 15 affected and 15 unaffected N_1_F_1_ mice were sent to the Australian Genome Research Facility (AGRF, Melbourne, VIC, Australia) where they were genotyped for 660 SNPs spaced at 5-Mb intervals throughout the genome using the iPLEX Gold method (Mendisco et al., 2011), the MassARRAY System (Sequenom, San Diego, CA) and an Autoflex MALDI-TOF mass spectrometer (Bruker, Billerica, MA). Mapmaker software (Lander et al., 1987) was used to calculate LOD scores, with the assumption that the phenotype was recessive. A Manhattan plot was drawn with Prism 6 for Mac OS X software.

N_1_F_1_ mice were genotyped for SNPs rs3142842, rs3676084, rs6197793 and rs13481117 using the Amplifluor SNPs HT genotyping system FAM-JOE (Merck Millipore, Kilsyth, VIC, Australia) and primers listed in supplementary material Table S1. DNA was vacuum-dried onto a 384-well plate. 5-μl PCR reactions containing 0.15 μM each forward primer, 2.25 μM reverse primer, 0.2 mM each dNTP (Merck Millipore), 1×FAM (Merck Millipore), 1×JOE (Merck Millipore), 1×S+ mix (Merck Millipore) and 0.05 μl titanium Taq DNA polymerase (Clontech Laboratories, Mountain View, CA) were added to the plate using an epMotion 5070 robot (Eppendorf South Pacific, North Ryde, NSW, Australia). PCR reactions were incubated at 94°C for 5 minutes, followed by 20 cycles of 94°C for 10 seconds, 55°C for 5 seconds and 72°C for 10 seconds, followed by 22 cycles of 94°C for 10 seconds, 55°C for 20 seconds and 72°C for 40 seconds, followed by a final extension at 72°C for 3 minutes. Fluorescence was measured with an infinite M200PRO plate reader (Tecan, Männedorf, Switzerland) using Magellan v7.1 software (Tecan). FAM was excited at 490 nm and emission measured at 530 nm. JOE was excited at 520 nm and emission measured at 560 nm. Results were visualized and genotypes assigned using assayauditorEP.xls (Merck Millipore) and excel v12.2.0 software (Microsoft, Redmond, WA).

### Mutation identification

The AGRF sequenced the exome of one *deaf14/deaf14* mouse using the 100803_MM9_exome_rebal_2_EZ_HX1 exome capture array (Roche Nimblegen, Madison, WI), TruSeq Sample Preparation Kit (Illumina, San Diego, CA) and HiSeq2000 Sequencing System (Illumina). Bioinformatic analysis of the raw sequence was performed by the Australian Phenomics Network (APN) using a custom workflow to align the sequence reads to the reference genome, filter the raw SNV calls and generate a list of candidate SNVs. Deep-sequencing datasets were deposited into the National Center for Biotechnology Information (NCBI) Sequence Read Archive (http://www.ncbi.nlm.nih.gov/sra, study accession number SRP020643).

The *Aspa* SNV was amplified using primers MC39 and MC40 (supplementary material Table S1) from DNA of two *deaf14* and two BALB/c mice. 25-μl PCR reactions contained 2 μl genomic DNA, 1×PCR buffer (Life Technologies, Mulgrave, VIC, Australia), 500 nM each primer, 200 nM dNTPs, 1.5 mM MgCl_2_ and 0.625 U Taq DNA polymerase (Life Technologies). Reactions were incubated at 94°C for 3 minutes then for 30 cycles of 94°C for 45 seconds, 55°C for 30 seconds and 72°C for 90 seconds, with a final extension at 72°C for 10 minutes. PCR products were visualized by agarose gel electrophoresis.

Unincorporated nucleotides and primers were removed with Exo-SAP-IT exonuclease (Affymetrix, Santa Clara, CA) according to the manufacturer’s instructions. 10-μl sequencing reactions containing 3 μl PCR product, 160 nM primer, 2 μl BigDye terminator v3.1 (Life Technologies) and 1 μl 5× buffer (Life Technologies) were incubated at 96°C for 2 minutes, followed by 25 cycles of 96°C for 10 seconds, 50°C for 5 seconds and 60°C for 4 minutes. Sequencing products were precipitated by addition of 75 μl 200 nM MgSO_4_ in 70% ethanol. After a 15-minute incubation, samples were centrifuged at 15,800 *g* for 15 minutes. Pellets were washed with 70% ethanol, dried at 37°C and submitted to the AGRF for capillary separation. Sequencing electropherograms were aligned using Seqman v10.1 software (DNASTAR, Madison, WI). Mice were genotyped for the *Aspa^deaf14^* mutation using the Amplifluor SNPs HT genotyping system FAM-JOE with the primers Aspa-F1, Aspa-F2 and Aspa-R (supplementary material Table S1).

### Histology

#### Cochlea

Mice were euthanized by intraperitoneal injection of 400 mg/kg body weight ketamine and 80 mg/kg body weight xylazine. After cessation of breathing, PBS was perfused through each animal via a cannula inserted into the left ventricle for 5 minutes, followed by 10% neutral buffered formalin for 5 minutes. Cochleae were dissected from the temporal bones and post-fixed for 1 hour at room temperature. Cochleae were washed in tris-buffered saline and decalcified in 10% EDTA for 5 days at 4°C with gentle rolling. Cochleae were oriented in 1% agarose in PBS in 10 mm×10 mm×5 mm cryomolds (Sakura Finetek, Torrance, CA) and paraffin-embedded. 2-μm sections were cut parallel to the modiolus using a microtome and stained with hematoxylin and eosin (H&E). Sections were imaged with a DM1000 compound microscope (Leica Microsystems, North Ryde, Australia) and DFC450 C camera (Leica Microsystems).

#### Brain

Mice were euthanized by carbon dioxide asphyxiation. Two methods of fixation were used prior to preparation of brain paraffin serial sections: 4% PFA perfusion fixation (as above) followed by submersion fixation for 2 days in 4% PFA and Bouin’s submersion fixation. For Bouin’s fixation, heads were dissected, removing skin, lower jaw, part of the snout and parietal bones and immersed in 40 ml Bouin’s fixative for 3 days. Following fixation, brains were dissected away from the remaining skull and meninges, returned to Bouin’s fixative for 2 days and then immersed in 70% ethanol. Approximately 650 serial sections were cut from each brain and stained with cresyl violet. Every ninth section was photographed (~90 sections per brain). Both fixation methods produced identical histological observation of spongiform encephalopathy in the *Aspa^deaf14/deaf14^* mice.

### Neuron and Schwann cell counting

ImageJ 1.46r software (imagej.nih.gov/ij) was used to determine the area of Rosenthal’s canal and to count cells. Cells with a large pink-stained nucleus, prominent nucleoli and a white halo were counted as neurons. Cells with a smaller purple-staining nucleus and without a white halo were counted as Schwann cells.

### Behavioral testing

Ten *Aspa^deaf14/deaf14^* mice (six female, four male) and nine BALB/c mice (four female, five male) underwent behavioral testing at the Melbourne Brain Centre (University of Melbourne, Parkville, VIC, Australia). These mice were 9- to 10-weeks old.

### Rotarod

Mice were placed on the Rotarod (Ugo Basile, Comerio, Basil, Italy) for four 5-minute trials, with a 1-hour inter-trial interval. During each trial, the Rotarod accelerated from 4 to 40 rpm over 5 minutes. The time taken for the mouse to fall off the Rotarod was measured in seconds.

### Open-field locomotor activity

Locomotor behavior was monitored using a TruScan photobeam activity monitoring system (Coulbourn Instruments, Allentown, PA). Animals were placed in the test arena for a 30-minute trial period and activity was detected electronically. The test arena was 40.6 cm wide×40.6 cm deep×40.6 cm high and encased by twin photo-optic arrays (sensor rings). The photo-optic beams were spaced 2.5 cm apart within each array, providing 1.3 cm spatial resolution. Distance travelled and number of moves were calculated using the TruScan software.

### Y-maze

Mice were placed in a custom-made Y-maze with three symmetrical 30-cm-long arms. Each of the arms had a distinctive visual cue at the end. The mouse was placed at the end of the home arm, facing away from the center, and was permitted to explore the home and familiar arms (a partition was used to block the novel arm) for 5 minutes. After a 2-hour interval, the mouse was once again placed in the maze, this time with the novel arm accessible. The time spent in each of the three arms was recorded over a 5-minute period.

### Light-dark test

The light-dark test was conducted using the TruScan locomotor system. A black Perspex insert (650 lux) was used to create a test field in which half the arena was dark and half was light. Each mouse was placed into the dark side of the chamber and permitted to move freely for 10 minutes. TruScan software was used to calculate the amount of time spent in the light versus the dark zones of the test arena.

### Western blotting

Mice were sacrificed by cervical dislocation and brains were snap frozen in liquid nitrogen. Each brain was dissolved in 5 ml lysis buffer containing 150 mM NaCl, 1% NP-40, 50 mM Tris pH 8.0 and protease inhibitors (88665, Pierce Biotechnology Inc., Rockford, IL) using a mortar and pestle on ice. Samples were agitated for 2 hours at 4°C then centrifuged at 13,400 ***g*** for 20 minutes. The concentration of protein in collected supernatant was determined using the QuickStart Bradford protein assay according to the manufacturer’s instructions (Bio-Rad Laboratories, Hercules, CA). 50 μg lysate was mixed with 2× reducing buffer (100 mM Tris pH 6.8, 4% SDS, 0.2 mg/ml bromophenol blue, 20% glycerol, 200 mM DTT) and denatured at 95°C for 5 minutes. Samples were electrophoresed on a 10% Bis-Tris gel (Life Technologies Australia Pty Ltd, Mulgrave, VIC, Australia) in 1× MOPS buffer (Life Technologies) then blotted onto Amersham Hybond-P PVDF membrane (GE Healthcare Australia Pty Ltd, Rydalmere, NSW, Australia) at 100 V for 1 hour in transfer buffer (200 mM glycine, 25 mM tris, 20% methanol). Blots were blocked in 5% skim milk powder in PBS for 1 hour then incubated with primary antibody in PBS 0.1% tween for 2 hours. Anti-ASPA antibody (ab154503, Abcam, Cambridge, UK) was used at 1:5000 and anti-actin antibody (ab95437, Abcam) was used at 1:500. Blots were washed for 5×10 minutes in PBS 0.1% tween then incubated in anti-rabbit IgG HRP (Cayman Chemical, Ann Arbor, MI) at 1:10,000 in PBS 0.1% tween for 1 hour. Blots were washed for 5×10 minutes in PBS 0.1% tween then incubated with Amersham ECL western blotting detection reagents (GE Healthcare) for 1 minute. Light emission was detected using an ImageQuant LAS 4000 (GE Healthcare).

### Statistics

ASR data was analyzed with one-way ANOVA to determine whether there was a significant difference between *Aspa^+/+^*, *Aspa^+/deaf14^* and *Aspa^deaf14/deaf14^* mice. When a significant difference was found, *Aspa^+/deaf14^* and *Aspa^deaf14/deaf14^* data were compared to *Aspa^+/+^* data with Dunnett’s multiple comparison test. All other data were analyzed with unpaired *t*-tests assuming equal variance. All statistical tests were performed using Prism 6 for Mac OS X software.

## Supplementary Material

Supplementary Material
