# Supplementary material for: A new mouse model of Canavan leukodystrophy displays hearing impairment due to central nervous system dysmyelination
Source: Dis Model Mech. 2014 Mar 28;7(6):649–57. doi: 10.1242/dmm.014605 (PMC4036472; doi:10.1242/dmm.014605)
Supplement: Supplementary Material [file supp_7_6_649__index.html]

A new mouse model of Canavan leukodystrophy displays hearing impairment due to central nervous system dysmyelination — Supplementary Material 

# A new mouse model of Canavan leukodystrophy displays hearing impairment due to central nervous system dysmyelination

## DMM014605 Supplementary Material

**Files in this Data Supplement:**

- **Supplementary Material**
