# Supplementary material for: A new mouse model of Canavan leukodystrophy displays hearing impairment due to central nervous system dysmyelination
Source: Dis Model Mech. 2014 Mar 28;7(6):649–57. doi: 10.1242/dmm.014605 (PMC4036472; doi:10.1242/dmm.014605)
Supplement: Supplementary Material [file supp_7.6.649_DMM014605.pdf]

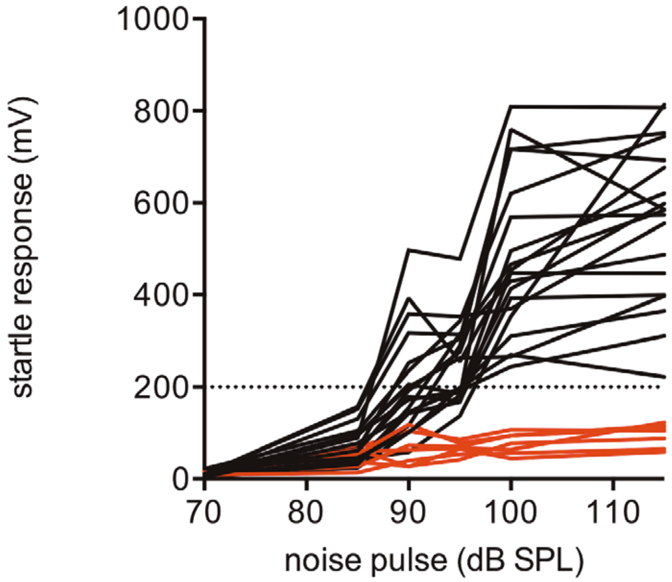

Supplementary Figure 1: Isolation of *deaf14* strain from ENU mutagenesis screen
Acoustic startle responses of 26 G_3 mice from the *deaf14* founder pedigree. Seven mice (red lines) displayed a response below 200 arbitrary units (dotted line). One of these mice was crossed to BALB/c and offspring intercrossed to found the *deaf14* line.

Supplementary Table 1: PCR primers

**Name**	**Sequence**
rs3142842-F1	GAAGGTCGGAGTCAACGGATTGTGGAGCTGAGAAGAGGAGG
rs3142842-F2	GAAGGTGACCAAGTTCATGCTGGTGGAGCTGAGAAGAGGAGA
rs3142842-R	ACCGCAGTGCATGGACTTT
rs3676084-F1	GAAGGTCGGAGTCAACGGATTTGCTCCTGCTTGTTAGCTGTCC
rs3676084-F2	GAAGGTGACCAAGTTCATGCTTCTGCTCCTGCTTGTTAGCTGTCT
rs3676084-R	GGTGGGTAGAAATGGAGAGAAT
rs6197793-F1	GAAGGTCGGAGTCAACGGATTAGTATTCCACCACTGGGCTGTA
rs6197793-F2	GAAGGTGACCAAGTTCATGCTTATTCCACCACTGGGCTGC
rs6197793-R	GCAGAACAGGAACCCTCAAT
rs13481117-F1	GAAGGTCGGAGTCAACGGATTTCAAGATTGTTCTCAGATCAGATTCC
rs13481117-F2	GAAGGTGACCAAGTTCATGCTGTCAAGATTGTTCTCAGATCAGATTCA
rs13481117-R	TCTACCCCACCCACCTATT
MC39	ACTTGCATGGCTCCATTACC
MC40	ACAGCTAGGGCTTGCCTACA
Aspa-F1	GAAGGTCGGAGTCAACGGATTCCACTCGTTCCATTGCCAAGTAA
Aspa-F2	GAAGGTGACCAAGTTCATGCTTCCACTCGTTCCATTGCCAAGTAT
Aspa-R	AGCAGGAAGTGTGACTTACCAA
